# Investigation and control of a suspected outbreak of carbapenem-resistant *Acinetobacter baumannii* nosocomial infections in the cardiovascular surgical ICU based on whole-genome sequencing

**DOI:** 10.3389/fpubh.2025.1730647

**Published:** 2026-01-13

**Authors:** Xiaochao Song, Zhewei Sun, Baixing Ding, Meijuan Jin, Jie Xu, Bo Yang

**Affiliations:** 1 Department of Infection Management, The First Affiliated Hospital of Soochow University, Suzhou, Jiangsu, China; 2 Institute of Antibiotics, Huashan Hospital, Fudan University, Shanghai, China; 3 Center of Clinical Laboratory, The First Affiliated Hospital of Soochow University, Suzhou, Jiangsu, China; 4 Department of Disinfection and Vector Control, Suzhou Center for Disease Control and Prevention, Suzhou, Jiangsu, China; 5 Suzhou Key Laboratory of Pathogenic Microorganisms for Emerging and Re-emerging Infectious Diseases, Suzhou Center for Disease Control and Prevention, Suzhou, Jiangsu, China

**Keywords:** carbapenem-resistant *Acinetobacter baumannii*, cardiovascular surgical ICU, outbreak, prevention and control, whole-genome sequencing

## Abstract

**Background:**

The treatment of carbapenem-resistant *Acinetobacter baumannii* (CRAB) infection faces great challenges. The purpose of this study was to investigate a suspected outbreak of CRAB infections in a cardiovascular surgical ICU to provide reference for clinical prevention and control.

**Methods:**

Patients infected with CRAB from 4 August to 29 August 2023 were included. Epidemiological investigation, environmental hygiene testing and pathogen testing were carried out. The whole genome sequencing and homology analysis were conducted to find out the possible infection source and transmission route, and corresponding control measures were taken in time.

**Results:**

Six patients developed CRAB lower respiratory tract infection in the short term, and five of them had similar drug sensitivity results. A total of 21 CRAB were detected from 146 environmental surfaces and the hands of medical staff. Phylogenetic tree and single nucleotide polymorphism (SNP) assessment indicated that the strain identified in Patient B and the simple resuscitation bag belonged to the same phylogenetic clade, but with more than 14 SNP differences. The other five patients and environmental strains constituted a separate clade, exhibiting closer phylogenetic relationships among certain patient strains and environmental strains, as well as among the environmental strains themselves. No new infection cases have occurred since the implementation of preventive and control measures.

**Conclusion:**

The CRAB infections in this department did not constitute a nosocomial infection outbreak; however, attention should be directed toward clonal transmission driven by mobile genetic elements. The urine bag drainage valve switch is identified as a potential transmission risk point that warrants special attention. It is imperative to enhance nosocomial infection prevention and control measures, including environmental disinfection, hand hygiene, and patient isolation.

## 1 Introduction

*Acinetobacter baumannii* is an opportunistic pathogen found in hospital environments and on human skin, in the respiratory and gastrointestinal tracts. It commonly causes pulmonary, bloodstream, and wound infections. In recent years, the extensive and improper use of broad - spectrum antibiotics has led to increasing microbial drug resistance, posing a major challenge to global public health (1, 2). The frequent and even abusive use of carbapenems has significantly increased the detection rate of CRAB. The data of China Antimicrobial Surveillance Network (CHINET) shows a rapid upward trend in CRAB detection rates (3). In the Mediterranean region, CRAB prevalence exceeds 90% (4), while it is lower in Europe (5), North America, and Latin America. Due to its high detection and resistance rates, the World Health Organization has listed it as a critical priority for new antibiotic development (6).

Cardiovascular surgical ICU patients are at higher risk for CRAB infection or colonization due to critical illness, low immunity, prolonged hospitalization, and extended antibiotic use. Frequent invasive procedures, patient care, transfers, and relatively concentrated bed placement further increase transmission risks, potentially causing cross-infection or outbreaks (7). CRAB is often multidrug-resistant or pandrug-resistant, making treatment complex and increasing patients’ hospital stay, medical costs, and mortality (8, 9). Thus, reducing CRAB infection and spread in ICUs is urgently needed. Previous studies mainly focused on ICU CRE infection outbreaks, with limited CRAB research based on molecular biology. This study investigated a CRAB hospital infection cluster in a cardiovascular surgical ICU and used whole-genome sequencing for molecular epidemiological and homology analysis to inform clinical treatment and infection control.

## 2 Materials and methods

### 2.1 Basic information of cluster cases of nosocomial infection

On August 25, 2023, the hospital’s Xinglin Real-Time Infection Monitoring System detected four patients in the cardiovascular surgical ICU who tested positive for CRAB. Subsequent screening identified two additional patients with CRAB on August 26 and 29, 2023. Based on clinical manifestations, imaging findings, and laboratory test results, all cases were diagnosed as CRAB lower respiratory tract infections. The diagnosis of nosocomial infection followed the “Diagnostic Criteria for Hospital Infections (Trial)” issued by the Ministry of Health (10). The diagnostic criteria for CRAB lower respiratory tract infections should simultaneously meet the following three items. 1. The patient must meet at least one of the following: (1) Fever; (2) Elevated total white blood cell count and/or neutrophil percentage; (3) New or progressive imaging abnormalities (pulmonary infiltrates or cavities). 2. At least two of the following criteria must be met: (1) New emergence of purulent sputum, change in sputum characteristics, increased respiratory secretions, or increased suctioning frequency; (2) New onset or worsening of cough, dyspnea, or tachypnea. (3) Wet rales or bronchial breath sounds. (4) Decreased oxygenation index (PaO₂/FiO₂), increased oxygen therapy requirement. 3. Isolation of CRAB as the predominant organism from sputum or lower respiratory tract specimens.

### 2.2 Epidemiological investigation

By reviewing clinical data and conducting on-site epidemiological investigations, we obtained preliminary information on the number of infections, the three-dimensional distribution (time, place, and person), infection status in previous and concurrent periods, and possible transmission routes. We defined cases as patients in our ward whose submitted specimens tested positive for CRAB. To expand the case search, we screened all other patients in the ward from August 1 to August 31 by collecting sputum and other relevant specimens in combination with clinical symptoms. Case verification was conducted based on clinical symptoms, physical signs, and laboratory findings.

### 2.3 Environmental hygiene investigation

On the day of sampling, a sterile phenol red dextrose broth medium was prepared. Each glass test tube was filled with 2.5 mL of phenol red dextrose broth, into which one meropenem susceptibility test disc (10 μg) and one vancomycin susceptibility test disc (30 μg) were placed for the initial screening of CRAB. For sampling environmental surfaces and palms, sterile flocked swabs were moistened with sterile saline and used to swab extensively in both horizontal and vertical directions. Samples were promptly transferred to culture tubes and incubated at 35 °C for 48 h in a constant-temperature incubator. Test tubes in which the phenol red broth retained its red color were considered negative, while those exhibiting a yellow-turbid appearance were selected for quadrant streaking on blood agar plates. Following incubation at 35 °C for 24–48 h, bacterial identification and antimicrobial susceptibility testing were conducted. CRAB was defined as *Acinetobacter baumannii* resistant to any carbapenem antibiotic.

A comprehensive survey was conducted across 146 sampling sites, which included environmental surfaces within the ICU ward, the soiled utility room, and doctors’ offices, as well as the hands of medical personnel. The distribution of these sampling sites was as follows: 47 sites were associated with bed units (including bed rails, bedside tables, bed foot tables, call buttons, height adjustment panels, and curtains) and medical equipment surfaces (such as ventilator controls, electrocardiogram monitors, infusion pump knobs, sphygmomanometers, and stethoscopes). Additionally, 7 sites were sampled from work attire, 7 sites from doctors’ offices and nurse station areas (including mouse and keyboard, desk, telephone, and medical record folders), and 5 sites from the soiled utility room (including washing machine handles and interior, bedpans, urinals, and mops). Furthermore, 28 sites were sampled from sink areas located in the ICU, soiled utility room, and doctors’ offices, while 12 sites were from the environment surrounding infected patients (including isolation gown surfaces, skin around endotracheal tubes, flooring, and air conditioning supply and return vents). Moreover, 21 sites were sampled from hand surfaces, 13 sites from urine-bag valve switch surfaces, and 6 sites from public items within the ward. Concurrently, oropharyngeal and nasopharyngeal swabs were collected from ward staff using the same culture medium for microbial screening.

### 2.4 Bacterial identification and antimicrobial susceptibility testing

The VITEK2-Compact automated microbial identification and antimicrobial susceptibility testing system (Bio-Merieux, France) and corresponding detection cards were used for the experiments. For drugs where the MIC range determined by the instrument did not meet the CLSI breakpoint criteria, disk diffusion or E-test strips were used. Antimicrobial susceptibility test results were interpreted according to the 2022 CLSI standards (11).

### 2.5 Whole-genome sequencing (WGS)

After pure culture, colonies were resuspended in buffer solution (20 mmol/L Tris–HCl, 5 mmol/L EDTA) and nucleic acids were extracted using the MagNA Pure 24 System. DNA concentration was quantified using the Qubit 3.0 Fluorometer. Genomic DNA was extracted, and libraries were constructed using the Illumina DNA Prep Tagmentation Kit. Sequencing was performed on the Illumina Miseq platform with a depth of ≥100 ×.

### 2.6 Single nucleotide polymorphism analysis and phylogenetic tree construction

Conduct quality control on raw reads using fastp to eliminate low-quality bases, resulting in clean reads. Subsequently, perform *de novo* assembly utilizing the assembly module of the EToKi tool, assigning strain numbers to the assembled contigs. Using the sequence from SAMN 40446890 as a reference, a maximum likelihood phylogenetic tree was constructed based on 2,691 core genome SNPs using EToKi (available at https://github.com/zheminzhou/EToKi). The resulting output was uploaded to the iTOL online platform (https://itol.embl.de) for graphical enhancement and annotation. Plasmid sequences were identified from the assembled genomes using the PlasT module of KleTy (DOI: 10.1186/s13073-024-01399-0; accessible at https://github.com/Zhou-lab-SUDA/KleTy). The ISFinder tool was employed to predict insertion sequences and transposons. Within R (version 4.4.3), the heatmap package was utilized to visualize the core SNP frequency matrix, while iTOL was used to visualize the phylogenetic tree and annotate sample information.

## 3 Results

### 3.1 Basic information of infected patients

During August 2023, the cardiovascular surgical ICU admitted 105 patients, among whom six developed CRAB lower respiratory tract infections, with an incidence rate of 5.71%. All six patients had undergone surgery and mechanical ventilation. Patient A was the first to test positive for CRAB in endotracheal aspirate (ETA) specimens on August 4. On August 22, three additional patients tested positive for CRAB in the ETA. Subsequent screening detected CRAB in two more patients on August 26 and 29. Of these six patients, four were male and two were female, aged between 34 and 69 years, with hospital stays ranging from 29 to 67 days (Table 1).

**Table 1:** General data of patients with CRAB infection in the cardiovascular surgical ICU.

Case	Gender	Age	Bed number	Date of detection	Sample type	ICU Admission time	Discharge time	Hospital admission diagnosis	Therapeutic antimicrobial agents
A	Male	36	EC12	2023/8/4	ETA	2023/7/22	2023/8/26	Type A aortic dissection	IPM combined with CIP
B	Male	69	EC11	2023/8/26	ETA	2023/8/16	2023/9/19	Tricuspid insufficiency	MEM combined with TGC
C	Female	63	EC5	2023/8/22	ETA	2023/8/16	2023/9/19	Type A aortic dissection	COL E combined with MEM
D	Male	34	EC14	2023/8/22	ETA	2023/8/15	2023/9/12	Type A aortic dissection	MEM combined with CIP
E	Female	64	EC2	2023/8/22	ETA	2023/8/11	2023/10/16	Left Atrial Appendage thrombus	MEM combined with TGC, CSL
F	Male	66	EC8	2023/8/29	sputum	2023/8/9	2023/9/14	Infective endocarditis	CSL

ETA, endotracheal aspirate; CSL, Cefoperazone/Sulbactam; IPM, Imipenem; MEM, Meropenem; CIP, Ciprofloxacin; TGC, Tigecycline; COL, Colistin.

### 3.2 Temporal and spatial distribution

**Figure 1:**
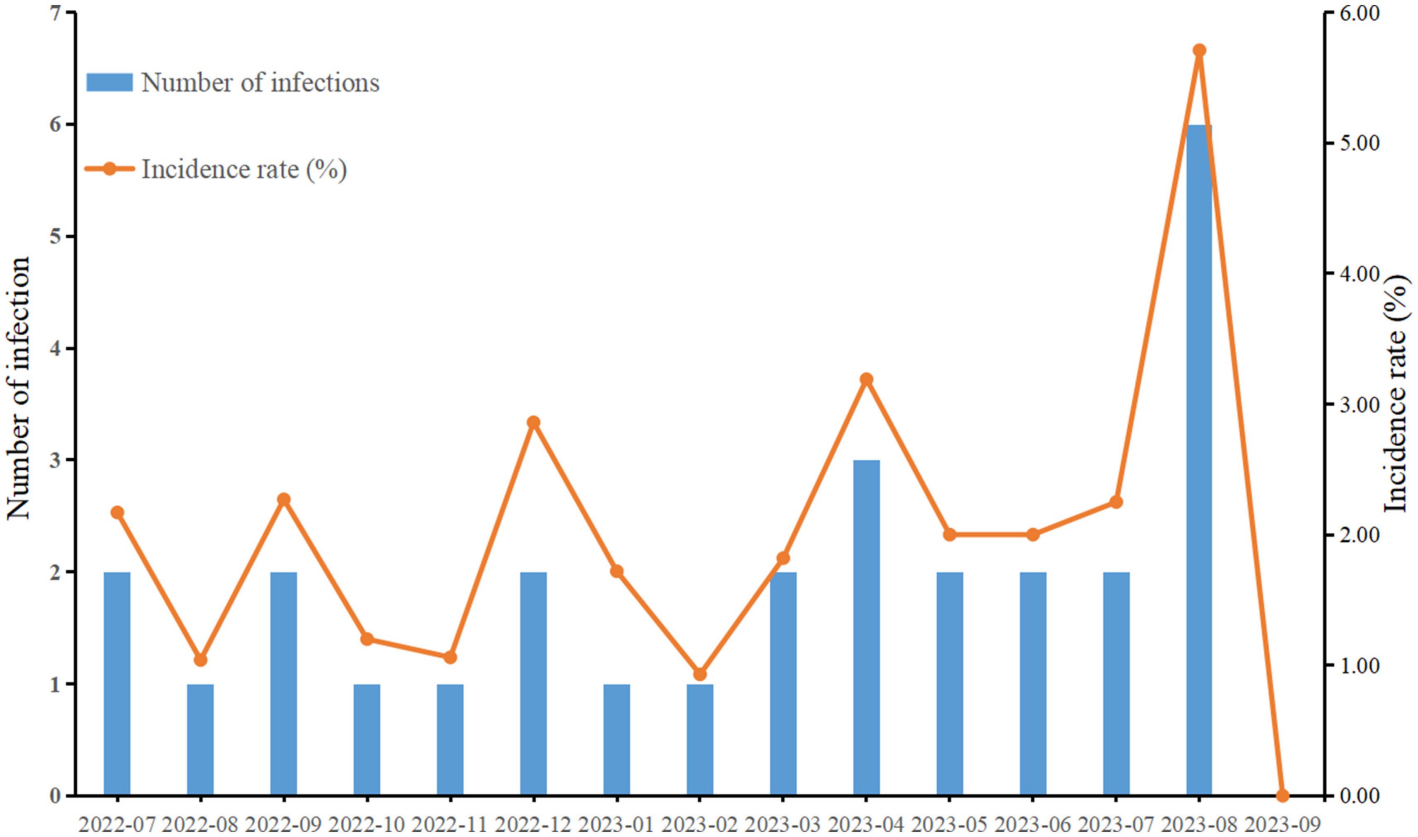
Status of CRAB nosocomial infections from July 2022 to September 2023.

**Figure 2:**
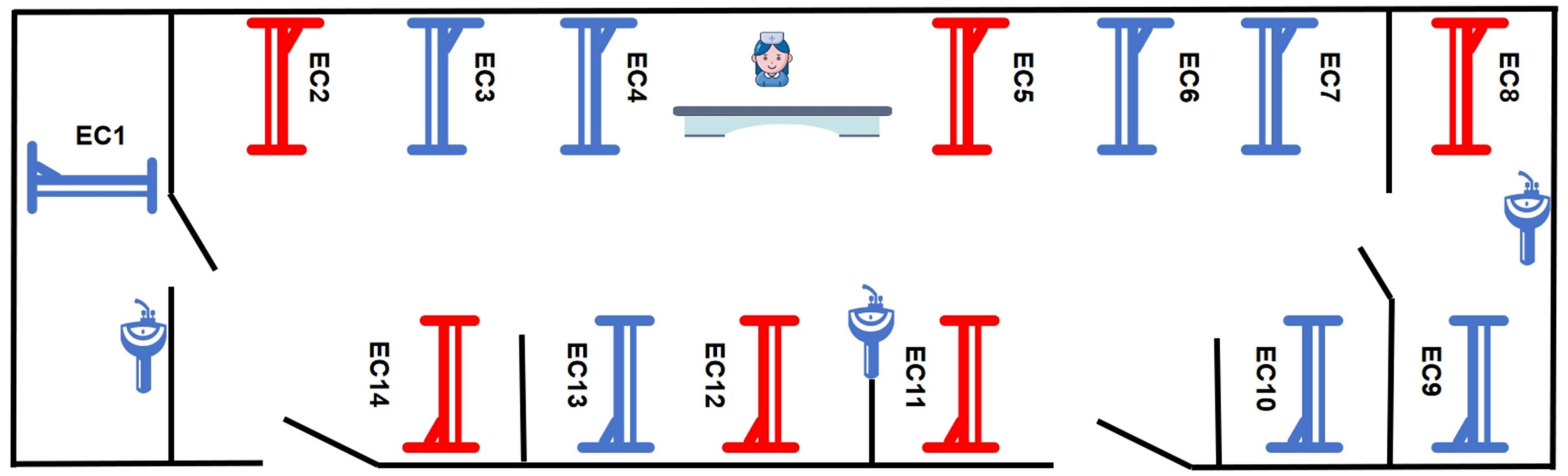
Spatial distribution of CRAB-infected patients in the cardiovascular surgery ICU. Red indicates the beds of infected patients, blue indicates beds of non-infected patients.

In 2022, among 96 patients, there was 1 case of CRAB lower respiratory tract infection, with an incidence rate of 1.04%. From July 1 to July 31, 2023, among 89 hospitalized patients, 2 cases of CRAB lower respiratory tract infection occurred, with an incidence rate of 2.25% (Figure 1). The six patients had overlapping hospitalization periods and were detected within a short time frame. The cardiovascular surgical ICU has 14 beds, including one single room, one double room, and one 11 - bed ward. The six patients were located in beds 2, 5, 8, 11, 12, and 14, with some beds in proximity. Five of the six patients were in the 11 - bed ward, while the remaining patient was in the double room (Figure 2).

### 3.3 CRAB detection and antimicrobial susceptibility

A total of 146 environmental and hand hygiene samples were collected, with 21 CRAB isolates detected, yielding a positive rate of 14.38%. During active patient care, 19 isolates were detected on bed units, medical equipment surfaces, ICU sink areas, and staff hands. Specifically, 2 isolates were found on the surfaces of urine bag drainage valve switches of infected patients, 3 on medical equipment surfaces, 1 on sphygmomanometers and stethoscopes, and 1 on isolation gowns. After environmental disinfection, 1 isolate was detected on the surfaces of bed units and medical equipment of non - infected patients, and another on the surface of a urine bag drainage valve switch. Notably, CRAB was not detected in any of the 38 oropharyngeal or nasopharyngeal swabs collected from 12 nurses, 12 doctors, and 2 orderlies within the ward.

CRAB strains from the environment showed resistance to most antibiotics. Only two strains had intermediate susceptibility to amikacin, and two were sensitive. In sputum samples, the strain from patient B had intermediate susceptibility to levofloxacin and was tigecycline - susceptible. The other five patients’ strains had consistent drug - susceptibility results and showed intermediate susceptibility to tigecycline (Table 2).

**Table 2:** Detection of CRAB and drug sensitivity analysis.

Sample ID	Isolation site	TZP	CSL	CTX	FEP	CAZ	IPM	MEM	LVX	CIP	GEN	AN	SXT	TGC
CDC321	Simple resuscitation bag	R	R	R	R	R	R	R	R	R	R	I	R	/
CDC319	ETA (Patient B)	R	R	R	R	R	R	R	I	R	R	/	R	S
CDC329	Hands (orderly)	R	R	R	R	R	R	R	R	R	R	R	R	/
CDC286	Bed unit (EC05)	R	R	R	R	R	R	R	R	R	R	R	R	/
CDC296	Emergency cart, treatment cart	R	R	R	R	R	R	R	R	R	R	R	R	/
CDC316	ETA (Patient D)	R	R	R	R	R	R	R	R	R	R	R	R	I
CDC292	Medical equipment surface (EC14)	R	R	R	R	R	R	R	R	R	R	R	R	/
CDC290	Urine bag drainage valve switch (EC05)	R	R	R	R	R	R	R	R	R	R	R	R	/
CDC287	Blood pressure cuff/stethoscope (EC05)	R	R	R	R	R	R	R	R	R	R	R	R	/
CDC293	Urine bag drainage valve switch (EC14)	R	R	R	R	R	R	R	R	R	R	R	R	/
CDC294	Faucet switch (ICU)	R	R	R	R	R	R	R	R	R	R	R	R	/
CDC301	Medical equipment surface (EC08)	R	R	R	R	R	R	R	R	R	R	R	R	/
CDC288	Medical equipment surface (EC05)	R	R	R	R	R	R	R	R	R	R	S	R	/
CDC303	Bed unit (EC14)	R	R	R	R	R	R	R	R	R	R	R	R	/
CDC283	Medical equipment surface (EC02)	R	R	R	R	R	R	R	R	R	R	R	R	/
CDC302	Bed unit, medical equipment surface (EC12)	R	R	R	R	R	R	R	R	R	R	S	R	/
CDC325	Isolation gown (EC14)	R	R	R	R	R	R	R	R	R	R	I	R	/
CDC326	Bed unit (EC10)	R	R	R	R	R	R	R	R	R	R	R	R	/
CDC323	Hands (orderly)	R	R	R	R	R	R	R	R	R	R	R	R	/
CDC304	Faucet outlet (ICU)	R	R	R	R	R	R	R	R	R	R	R	R	/
CDC320	Sputum specimen (Patient F)	R	R	R	R	R	R	R	R	R	R	/	R	I
CDC314	ETA (Patient A)	R	R	R	R	R	R	R	R	R	R	/	R	I
CDC315	ETA (Patient E)	R	R	R	R	R	R	R	R	R	R	/	R	I
CDC322	Work clothes (orderly)	R	R	R	R	R	R	R	R	R	R	R	R	/
CDC327	Urine bag drainage valve switch (EC06)	R	R	R	R	R	R	R	R	R	R	R	R	/
CDC318	ETA (Patient C)	R	R	R	R	R	R	R	R	R	R	R	R	I
CDC324	Hands (nurse)	R	R	R	R	R	R	R	R	R	R	R	R	/

ETA, endotracheal aspirate; TZP, Piperacillin/Tazobactam; CSL, Cefoperazone/Sulbactam; CTX, Cefotaxime; FEP, Cefepime; CAZ, Ceftazidime; IPM, Imipenem; MEM, Meropenem; LVX, Levofloxacin; CIP, Ciprofloxacin; GEN, Gentamicin; AN, Amikacin; SXT, Trimethoprim/Sulfamethoxazole; TGC, Tigecycline; R, resistance; I, intermediate; S, susceptible; “/,” no drug sensitivity test was performed.

### 3.4 Whole-genome sequencing and phylogenetic analysis

**Figure 3:**
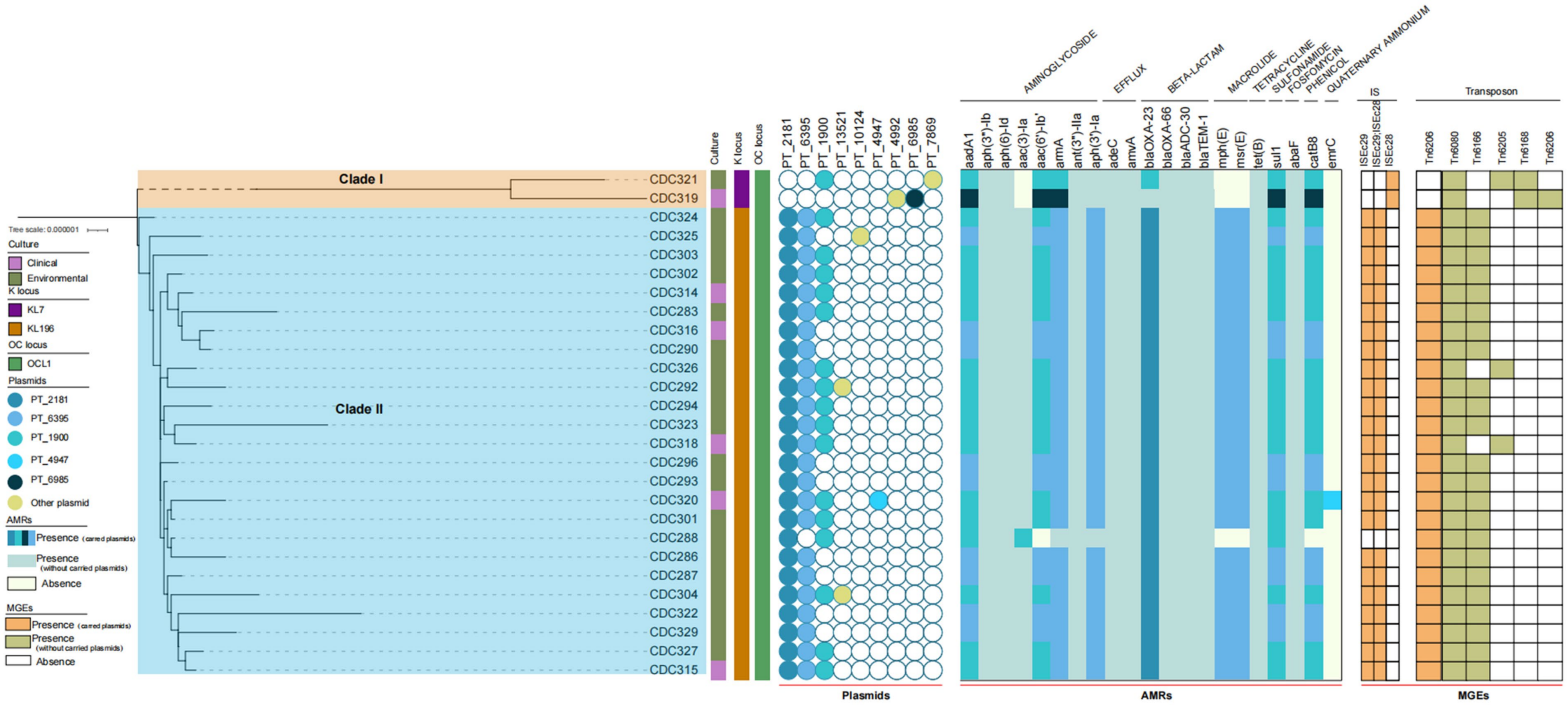
Phylogenetic tree and the molecular characteristics of 27 CRAB isolates based on core genome SNPs. KL: Capsular polysaccharide locus; OCL: Lipooligosaccharide outer core biosynthesis locus; AMRs: Antimicrobial resistances; MGEs: Mobile genetic elements.

Two main phylogenetic clades were identified: one clade included the isolate from patient B and the simple resuscitation bag, and the other clade included all other isolates, indicating a close genetic relationship between the two groups (Figure 3). Multilocus sequence typing (MLST) revealed that all isolates belonged to sequence type ST2. All isolates were identified as lipopolysaccharide outer core locus 1 (OCL 1), with only the isolates from patient B and the simple resuscitation bag identified as capsular polysaccharide locus 7 (KL 7), while all other isolates were KL 196. All strains harbor the antibiotic resistance genes *bla*OXA-66*, bla*OXA-23*, bla*ADC-30*, bla*TEM-1*, aadA1, aph(3″)-Ib, aph(6)-Id, armA, ant(3″)-IIa, aph(3″)-Ia, adeC, amvA, sul1* and *tet(B)*. Most strains carried plasmids of types PT_2181, PT_6395, and PT_1900, along with transposons Tn6166, Tn6080, and Tn6206, as well as insertion sequences ISEc29 and ISEc28.

### 3.5 SNP difference matrix analysis

**Figure 4:**
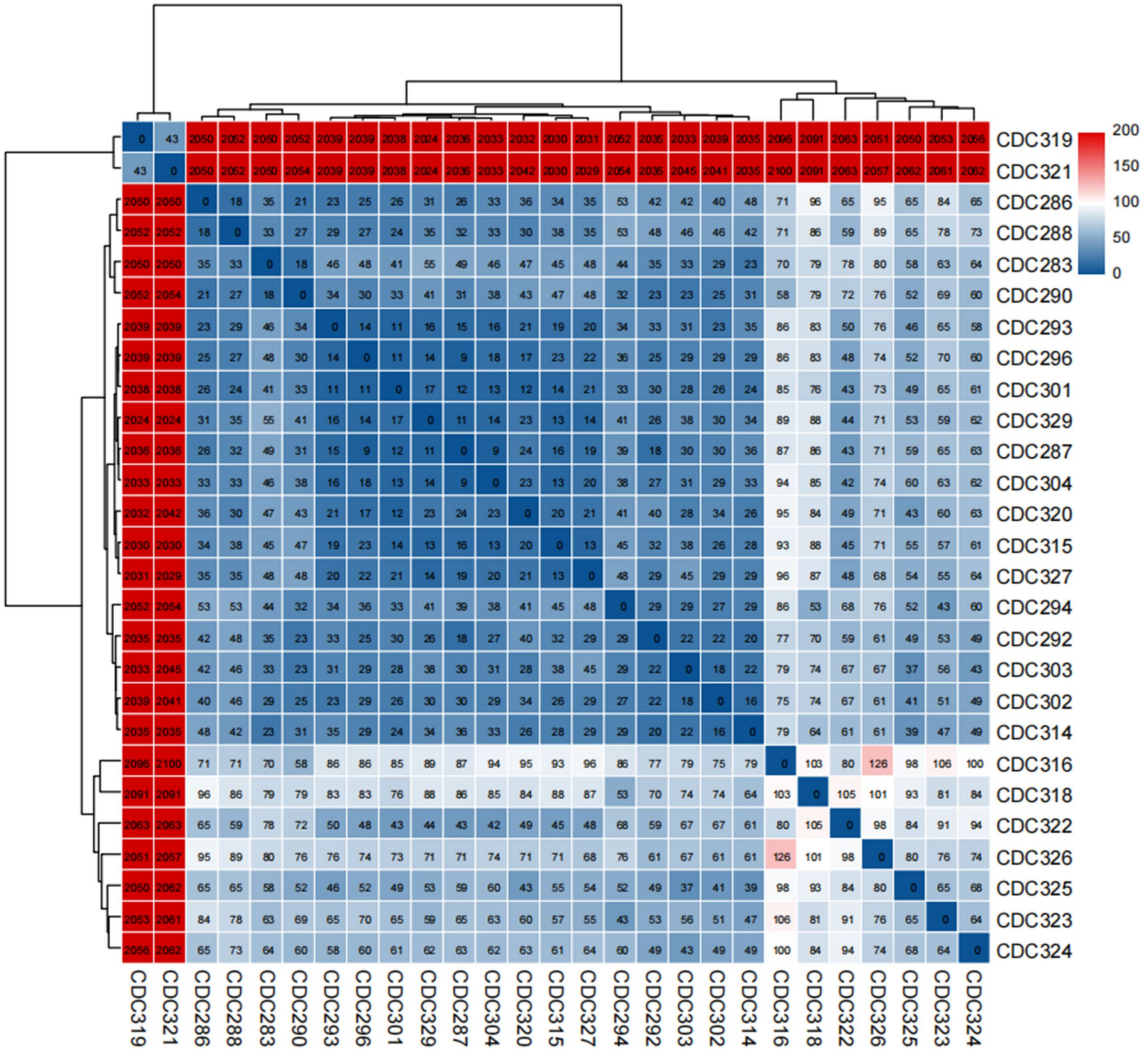
Heatmap of 27 CRAB isolates based on core genome SNPs. The gradation of blue and red color intensity represents the degree of core genome SNP differences among different samples.

High-quality SNPs (with a frequency and base quality of over 20%) were detected between every pair of strains. SNP differences of < 14 indicated homology (12). Patient B and the simple resuscitation bag had 43 core genome SNP differences, 2024 ~ 2063 differences from other environmental isolates, and 2030 ~ 209 differences from other patients. Differences in SNPs among other patients ranged from 20 to 103. Five patients exhibited SNP differences ranging from 12 to 126 when compared to environmental isolates. Specifically, Patient F (CDC320) and the instrument surface (CDC301) showed 12 SNP differences. Patient E (CDC315) demonstrated 13 SNP differences with the faucet outlet (CDC304), the urine bag drainage valve switch from another patient’s urine collection bag (CDC327), and the hand of a support staff member (CDC329). With the exception of the simple resuscitation bag, all other CRAB strains detected in the environment exhibited SNP differences ranging from 9 to 98 per pair. Among these, seven pairs of isolates had SNP differences of less than 14 (Figure 4).

### 3.6 Infection control measures and outcomes

During the investigation, we promptly guided the implementation of the following hospital infection control measures: (1) assigning dedicated caregivers and specialized medical teams; (2) promoting hand hygiene through hand disinfection and enhancing hand hygiene training and monitoring for orderly and medical staff; (3) replacing ICU sink faucets with touch-free ones and strengthening pipeline disinfection; (4) intensifying cleaning and disinfection of environmental surfaces and medical equipment, increasing the frequency to three times daily; and (5) centrally washing orderlies’ clothes daily, increasing the reserve of isolation gowns, and providing training and assessment on the proper use of protective gear. Following these enhanced interventions, no new CRAB cases were identified in September, and the CRAB cluster was successfully controlled.

## 4 Discussion

CRAB infections are globally prevalent, with varying levels of prevention and control across regions and institutions, posing a significant challenge. The isolation and infection rates of CRAB are particularly severe among ICU patients, making it the leading pathogen in hospital-acquired infections (13). The respiratory tract is the most common site of CRAB detection, accounting for up to 70% of infections (14). In our study, six cases of CRAB lower respiratory tract infections occurred in a cardiovascular surgical ICU within a short period, suggesting a potential hospital outbreak. Upon investigation, all infected patients had high-risk factors for CRAB infection, including central venous catheterization, mechanical ventilation, nasogastric tube placement, and surgery (15). All six patients underwent fiberoptic bronchoscopy with bronchoalveolar lavage. Records of disinfection efficacy for the two bronchoscopes used showed no bacterial overgrowth or detection of pathogenic organisms, ruling out transmission via this route. All patients had overlapping hospital stays, occupied concentrated bed spaces, and lacked dedicated medical and nursing teams, creating potential for contact transmission, droplet transmission, and airborne transmission.

CRAB can persist for extended periods on surfaces in healthcare settings (16), with a higher prevalence in ICU environments. The investigation revealed a high detection rate in the Cardiovascular surgical ICU environment at this hospital. CRAB was more frequently identified in the environments surrounding infected patients, with a notable detection rate also observed in the vicinities of other patients and on communal objects. It is recommended that the frequency and extent of environmental cleaning and disinfection be enhanced (17). Most environmental CRAB isolates have similar antibiotic resistance profiles, with only a few strains sensitive to amikacin. Except for patient B, the antibiotic susceptibility results of the strains from patients A, C, D, E, and F were consistent and similar to most environmental isolates. The majority of hospitalized patients undergo closed suctioning procedures. Notably, no CRAB isolates were detected in the air conditioning supply or return vents. This observation suggests that, in addition to endogenous CRAB infections, exogenous infections resulting from contact transmission may also be present. Variations in resistance genes and resistance phenotypes of CRAB have been observed within our department. Therefore, treatment regimens should be tailored to incorporate the department’s specific bacterial epidemiological characteristics to ensure rational initial empirical therapy (14, 18).

On a global scale, ST2-type CRAB strains that produce the acquired OXA-23 carbapenemase are predominant. Consistent with domestic molecular epidemiological characteristics (19), the primary mechanism of carbapenem resistance in CRAB isolates from this city and other regions within the province is attributed to the presence of *bla*OXA-23 and *bla*OXA-66 genes (20, 21). In this study, all CRAB isolates obtained from patients and environmental sources were classified as ST2 and were found to produce both OXA-23 and OXA-66 carbapenemases. The six patients were categorized into two distinct potential outbreak clusters, with each pair demonstrating more than 14 core genomic SNPs between their detected strains. This finding suggests a low probability of nosocomial cross-infection events. Antimicrobial resistance genes can be transmitted vertically via chromosomes or horizontally among different species and strains through plasmids, integrons, transposons, and other mobile genetic elements (22). In this study, CRAB strains predominantly shared multiple mobile genetic elements, such as plasmids and transposons, indicating a clonal spread primarily facilitated by horizontal gene transfer rather than a hospital-acquired outbreak (23, 24). However, we identified transmission chains between patients and their environment, as well as among different environmental surfaces. The SNP differences observed in CRAB-infected strains and surfaces of equipment, sphygmomanometer cuffs/stethoscopes, urine bag valve switches, faucet outlets, ward resuscitation carts/treatment carts, and healthcare workers’ hands were less than 14. Consequently, contaminated environmental surfaces, medical devices, healthcare workers’ hands, and individuals infected or colonized with CRAB present a risk of cross-transmission, potentially leading to nosocomial infection (25). Accordingly, contact isolation measures should be promptly implemented, especially for susceptible patients with indwelling catheters, open wounds, or weakened immunity (26). Staff should be rationally allocated, with grouped nursing care and diagnostic/treatment care scheduled last (27).

Studies have shown that sinks are high-risk areas for MDRO detection in ICUs (28). In this survey, the sinks and surrounding floors in the ICU, utility room, and doctors’ office tested negative, but the faucet and outlet of the ICU sink tested positive. The touch - operated sink faucet may be contaminated when turned on after patient care and then contaminate the outlet. Additionally, CRAB was detected on the hands of orderlies and nurses during work, but not after hand hygiene. Therefore, hand hygiene, preferably with hand disinfection, is recommended, and touch - free faucets should be used if hand - washing is necessary. Notably, CRAB was detected on the urine bag drainage valve switches of both infected and non - infected patients, indicating low hand hygiene compliance and accuracy among caregivers. Unlike high - touch environmental surfaces, these switches are usually not disinfected daily and pose a potential infection spread risk if hand hygiene is inadequate. Moreover, CRAB was found on orderlies’ uniforms and bedside isolation gowns of infected patients. This suggests that proper wearing and removal of isolation gowns during invasive procedures and extensive patient contact are essential (29). We recommend centralized daily washing and disinfection of orderlies’ uniforms and reinforced training on isolating gowns.

## 5 Conclusion

In conclusion, the integration of epidemiological data with molecular biological characterization enhances the identification of potential infection sources and transmission pathways. Although a definitive transmission chain among hospital-acquired CRAB infection cases has not been established, it is crucial to address the spread of resistance and environmental contamination resulting from horizontal gene transfer. It is imperative to implement targeted, cluster-based interventions to control infection sources and disrupt transmission routes, while simultaneously providing medical treatment informed by molecular epidemiological characteristics. This study is subject to several limitations: the absence of patient and environmental isolates prior to August precluded a comprehensive spatiotemporal epidemiological analysis; additionally, constraints related to sequencing depth and funding restricted analyses of resistance islands and associated accessory genomes, highlighting the need for further in-depth investigation in future research.

## Data Availability

The datasets presented in this study can be found in online repositories. The names of the repository/repositories and accession number(s) can be found at: https://www.ncbi.nlm.nih.gov/, PRJNA1394720.
